# Superharmonic Resonance in Carbon-Black-Filled Rubber by High-Frequency DMA

**DOI:** 10.3390/polym11101653

**Published:** 2019-10-11

**Authors:** Imran Hussain Syed, Jorge Lacayo-Pineda

**Affiliations:** Continental Reifen Deutschland GmbH, Jaedekamp 30, 30419 Hannover, Germany; jorge.lacayo-pineda@conti.de

**Keywords:** nonlinearity, superharmonic resonance, HF DMA

## Abstract

A systematic study of several SBR compounds filled with carbon black of various grades were analysed with the high-frequency Dynamic Mechanical Analyzer (HF DMA) in order to quantify the degree of nonlinearity induced by fillers in rubber compounds. These filler grades indirectly reflect different degrees of microdispersion, which seems to be the main influence on the superharmonic resonance phenomenon observed in HF DMA. This statement arises from the comparison of the microdispersion observed in TEM images. In the second part of the paper, a model compound filled with carbon black is enhanced with a standard reinforcing resin, which leads to a more compact filler network. This induces a higher superharmonic resonance response as well as a higher transmissibility behaviour.

## 1. Introduction

Fillers are introduced into rubber compounds to improve their mechanical properties such as elongation at break, shore hardness, and abrasion [1,2,3]. It is known from Fourier Transform (FT) rheology that carbon black enhances the nonlinear mechanical behaviour of the compound. The nonlinearity here is referring to the appearance of higher harmonics within the DMA response signal for a sample that is under defined deformation conditions. Nonlinearity is known to be quantified by the ratio of the third to the first harmonic amplitude, I(3/1) in FT Rheology [4,5,6,7,8]. While FT rheology is a strain dependent analysis of the Fourier response, the high-frequency Dynamic Mechanical Analyzer (HF DMA) allows for a resonance-based, frequency dependent analysis of the Fourier response. The outcome of this frequency analysis is the appearance of the vibrational superharmonic resonance.

Vibrational resonance was first reported by Landa and McClintock [9] in order to describe a nonlinear signal response which is weakly perturbated by a secondary, significantly weaker oscillator into the system. Following this work, Yang et al. [10] observed higher resonance response from the same system. In order to describe this behaviour, the terms vibrational superharmonic resonance (VsupR) and vibrational subharmonic resonance (VsubR) were proposed to represent the presence of higher-order and lower-order resonances, respectively.

In the previous investigation, a superharmonic resonance phenomenon was observed in rubber compounds using the Fourier analysis of the resonance peak from HF DMA [11]. There exists a transition between polymer-related nonlinearity and filler-induced nonlinearity. This transition coincides with the percolation threshold of the filler network and gives a hint on the origins of the observed superharmonic resonance. The present work is aimed at elucidating the correlation between the filler network and the superharmonic resonance.

In another investigation, it was concluded that novolac-type phenolic resin changes the interfacial properties of the carbon black (CB) filler network [12]. This was concluded based on thermomechanical as well as microscopical measurements on filled isoprene rubber compounds with various resin concentrations. The reinforcing resin reduces the percolation threshold of filled compounds by creating interfaces between the carbon black aggregates.

The current investigation is divided into two parts; the influence of polymer types and carbon black grades, and the influence of reinforcing resins. In the first part, an isoprene rubber (IR) filled with N339 carbon black is compared with a solution-polymerized styrene-butadiene rubber (SSBR)-type of the same filler grade. Then, using the SSBR compound as a base, three different filler grades, N121, N339, and N550 are used to compare the filler grade influence on the superharmonic resonance. The second part of the paper aims to further elucidate the superharmonic resonance by introducing reinforcing resin which has been proven to reduce the percolation threshold by modifying the carbon black interfaces.

## 2. Materials and Methods

The main tool for this investigation is the high-frequency DMA, VHF104 by Metravib, Lyon, France. The general concept of the device is the measurement of the resonance peak on a system comprising of the sample and two metal cylinders; one acting as the excitation base and the other as the preload force onto the sample. The fundamental advantage of this device is the ability to directly measure the mechanical response of a material up to 10 kHz, as opposed to other traditional DMA devices which typically have a maximum frequency of 100 Hz. In terms of absolute deformation amplitude, the latter can achieve values of 9 mm at low frequencies (e.g., DMA Gabo Eplexor 2000N^®^, Netzsch, Alhden, Germany), while the high-frequency DMA is able to reach values of around 300 μm. This value however is highly dependent on the applied top mass of the system.

This DMA is a type of forced vibration resonant system [13], which indicates that the experimental setup must be calibrated in terms of sample geometry and added mass in order to obtain the material resonance in the desired frequency window. The resonance frequency $f_{o}$ in tension–compression mode can be estimated with the following equation:

(1) $$f_{o}=\frac{1}{2\pi }\sqrt{\frac{E\cdot S}{l\cdot M}},$$

where *E* is the Young’s modulus in N/m${}^{2}$, *S* is the cross-sectional contact area in m${}^{2}$, *l* is the height of the sample in m, and *M* is the added top mass in kg.

The VHF104 is able to operate at temperature ranges between $-50{\;}^{\circ }$C to $100{\;}^{\circ }$C, however, the focus of the present work will be limited to ambient conditions. The excitation displacement was set to 0.1 mm, however, when the value cannot be achieved after certain frequencies, a maximum excitation acceleration of 200 mm/s${}^{2}$ is used instead due to the limitations of the oscillator acting on the excitation base. Therefore, a correction procedure is applied in the evaluation in the next section. Finally, the tension–compression deformation modes were selected due to the relative simplicity of the sample geometry.

For an independent determination of the percolation threshold, dynamic strain-dependent measurements were performed on the Rubber Process Analyzer RPA 2000 by Alpha Technologies, Hudson, NY, USA. The applied strain amplitudes were chosen from 0.3% up to 100% at $70{\;}^{\circ }$C.

The transmission electron microscopy (TEM) analysis was performed on a JEM1400 Instrument (Jeol, Tokyo, Japan) using a voltage of 100 kV. In order to investigate the CB network structure, samples were measured in the bulk state. Thin slices of approximately 60 nm were prepared by using a cryo-ultramicrotome (Leica EM UC6/EM FC6) equipped with a diamond knife.

The samples chosen for the investigation are solution-polymerized styrene-butadiene rubber (SSBR) compounds with three different grades of carbon black; N121, N339, and N550, at various concentration levels. Additionally, isoprene rubber (IR) compounds with various concentrations of CB N339 and reinforcing resin were investigated. These recipes are summarised in Table 1 and Table 2, respectively.

## 3. Results and Discussion

### 3.1. The Superharmonic Resonance

The analytical approach for the extraction of superharmonic resonance values has been discussed elsewhere [11]. The first vibrational mode is fitted with the linear transmissibility formula, which is then used to normalise the 2nd vibrational mode. The resonance peak of the normalised 2nd vibrational mode is defined as the 1st superharmonic resonance. In the current investigation, the term is simplified to superharmonic resonance since higher superharmonic resonances are more prone to measurement noise.

In the same investigation, the amount of N339 carbon black was varied in SSBR and measured with HF DMA. Two distinct nonlinearities were observed; the nonlinearity originating from the polymer, and the filler-induced nonlinearity. Above the percolation threshold, the latter becomes more dominant than the former. In the aforementioned investigation, the applied strain was assumed to be constant throughout the measurement range. This assumption was sufficient within the experimental setup as to understand the nonlinear behaviour observed. Increasing the level of compound complexity requires a more rigorous consideration of the strains.

**Figure 1:**
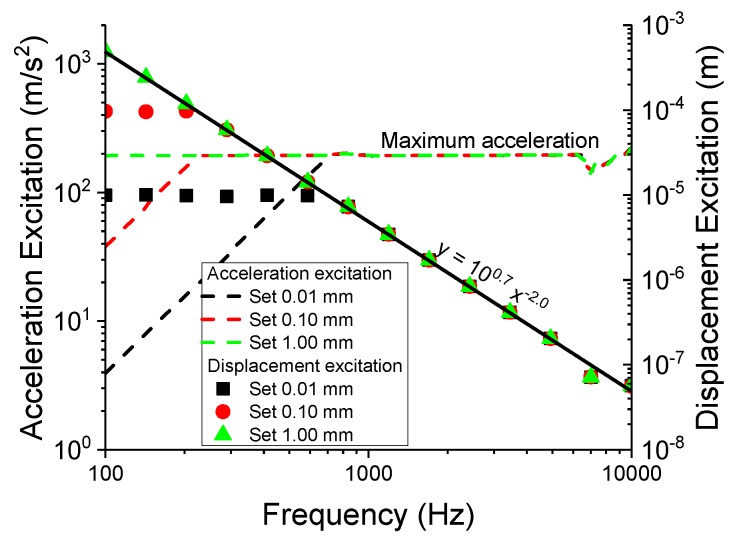
The excitation relation between the displacement and acceleration.

When considering different compounds, it is important to note that the excitation input of the experiment is a function of both amplitude and frequency. There is, however, an instrumental limitation on the excitation since a substantial amount of power would be required to have a large amplitude at higher frequencies. As shown in Figure 1, the displacement excitation is reduced at higher frequency ranges. When the desired excitation amplitude cannot be achieved, a constant base acceleration of 200 m/s${}^{2}$ is used. The drop in amplitude with respect to frequency can be formulated as follows:

(2) $$Excitation\;displacement,x_{input}=10^{0.7}\cdot frequency_{input}^{-2}.$$

Since the resonance frequency $f_{o}$ is shifted due to the difference in sample modulus, the sample does not experience the same level of excitation. This can be accounted for by using $f_{o}$ as a prefactor for the subsequent normalised superharmonic resonance (nSHR). Hence, for simplicity, a new term is introduced to accommodate both the superharmonic resonance as well as the aforementioned corrections:

(3) $$normalised\;superharmonic\;resonance,nSHR=\frac{\left(f_{o}\right)\left(1st\;superharmonic\;resonance\right)}{resonance\;amplitude}.$$

**Figure 2:**
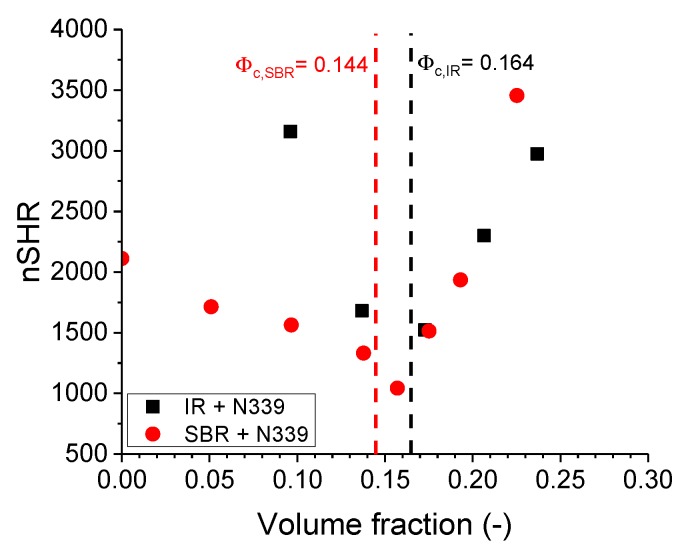
The normalised superharmonic resonance for carbon black (CB)-filled styrene-butadiene rubber (SBR) and isoprene rubber (IR) compounds. The percolation thresholds were obtained by Rubber Process Analyzer (RPA).

The nSHR values between SBR-filled compounds and IR-filled compounds are shown in Figure 2. Above the percolation threshold, the slopes of nSHR for both polymer compounds are similar, and henceforth, indicates a similar filler-induced nonlinearity of the CB. Below the percolation threshold however, the gradient is different, hence, an indication of a polymer-dependent nonlinear response.

### 3.2. Carbon Black Grades

The three variants of CB grades are known to have different filler structure and size. It is also known that the CB structure plays an important role in the nonlinearity of the compound within FT Rheology [14]. Therefore, a similar approach is used in the present work to decouple the filler influence and the polymer matrix response.

**Figure 3:**
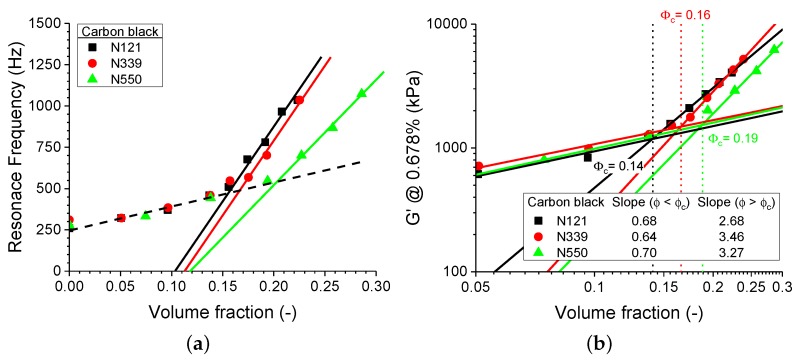
The (**a**) resonance frequency and (**b**) the corresponding storage modulus at low strain of the solution-polymerized styrene-butadiene rubber (SSBR) compounds, as described in Table 1. The dotted lines in (**b**) indicate the corresponding percolation thresholds obtained by RPA measurements.

When comparing the resonance frequency in HF DMA with respect to the CB volume fraction, as shown in Figure 3, the onset of the filler network reinforcement as indicated by the percolation threshold can be estimated in analogy to the RPA whereby the deviation of the hydrodynamic reinforcement of the sample determines the percolation threshold. It should be noted from Equation (1) that the resonance frequency is a function of both modulus and geometry. Nevertheless, when comparing the same geometry, the resonance frequency shows a similar strain dependency with respect to CB volume fractions, such is the case for the dynamic strain sweep measurements from the RPA.

**Figure 4:**
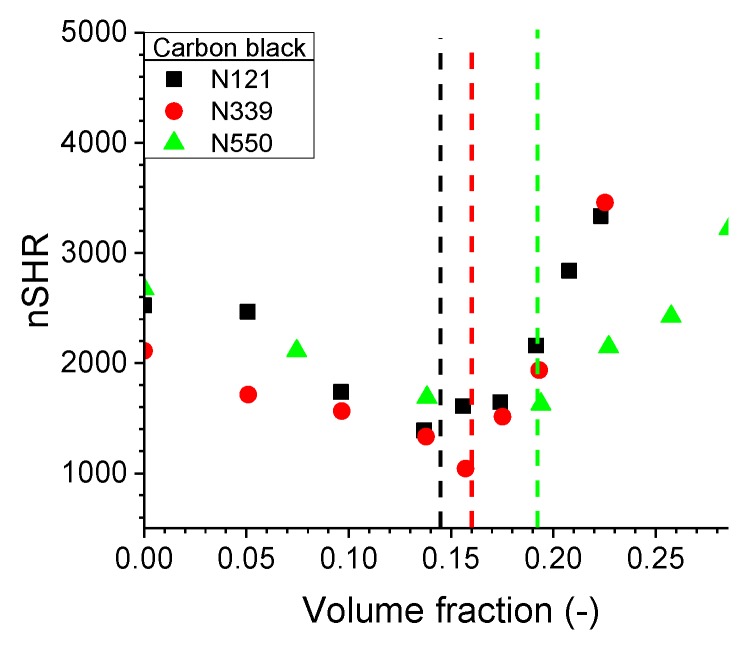
The normalised superharmonic resonance (nSHR) values for three carbon black grades; N121, N339, and N550 within a SSBR matrix. The dashed vertical lines represent the percolation threshold obtained by RPA for each compound.

**Figure 5:**
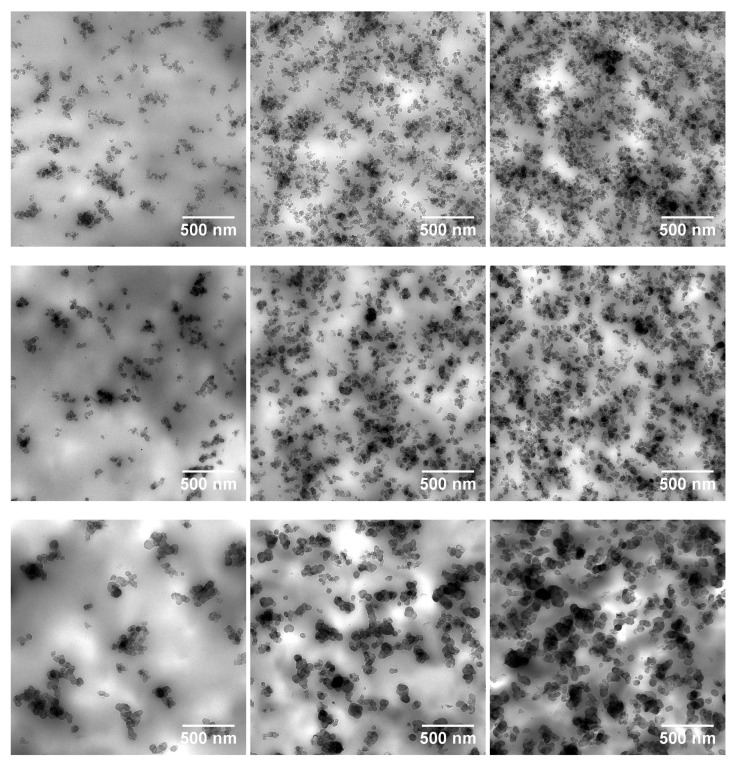
TEM images for N121 (**top row**), N339 (**middle row**), and N550 (**bottom row**) in SSBR compounds with filler concentrations below, around, and above percolation threshold.

The normalised superharmonic resonance as a function of CB volume fraction is shown in Figure 4. Similar to Figure 3, the onset at the percolation threshold can be observed. For N121 and N339 compounds, the nSHR value as an indicator of nonlinearity shows a similar CB volume fraction dependency, while the N550 compound shows a much weaker dependency. One plausible explanation of this behaviour is the relatively poor microdispersion of N550 present in the rubber matrix. Images from TEM in Figure 5 seem to justify the hypothesis, especially when compared to the other two carbon black grades. It is possible that the filler particles are scattering the mechanical wave propagation through the sample from the input to the output sensor. Above the percolation threshold however, a secondary wave propagation is induced by the filler structure due to the large modulus difference between the viscoelastic matrix and the filler network. This implies that the slope of nSHR might be an indication of filler distribution within the compound.

### 3.3. Reinforcing Resin

In the previous investigation, it was concluded that novolac-type phenolic resin changes the interfacial properties of the CB filler network [12]. This resin-mediated filler network enhances the mechanical properties of the material. This was inferred when the percolation threshold of the polymer was reduced with the introduction of resin as well as the higher mechanical softening behaviour with respect to the applied strain deformation and temperature dependency. Therefore, the filler-induced nonlinearity should be affected by the modification of the filler network interphase by resin.

**Figure 6:**
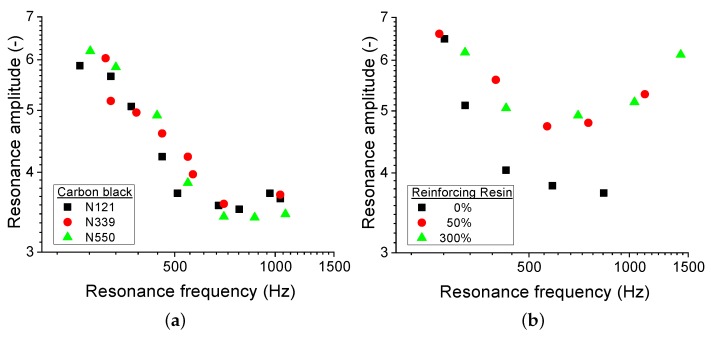
The resonance amplitude as a function of resonance frequency for (**a**) different carbon black grades within a SSBR matrix without resin and (**b**) carbon-black-filled IR with three resin concentrations; 0.0, 1.4, and 9.2 parts per hundred rubber.

The resonance amplitude is inversely proportional to the damping coefficient. Looking at the resonance amplitude of the first vibrational mode in Figure 6b, the damping coefficient is seen to be reduced for the compound with substantial amount of resin. This means that the polymer has a stronger elastic behaviour with a higher amount of filler and resin combinations. This behaviour was not observed in the aforementioned CB variation studies, as indicated in Figure 6a. In other words, the resin-mediated compounds are able to channel the mechanical wave more effectively through the sample without heavy signal attenuation.

**Figure 7:**
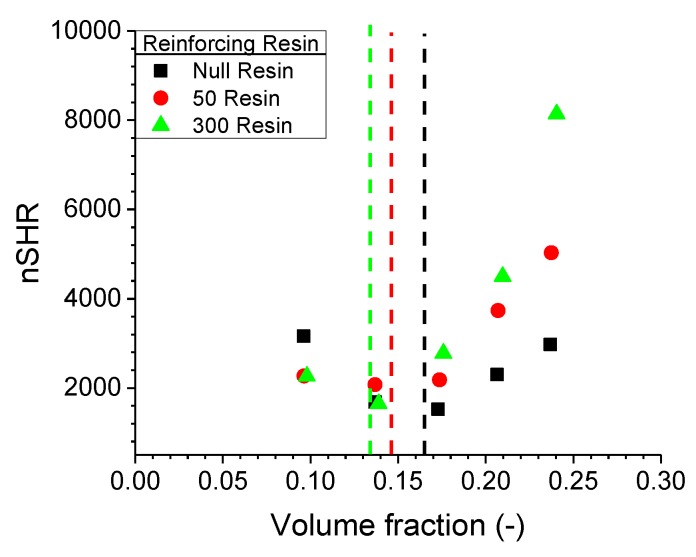
The nSHR values for the three resin concentrations in a N339-filled IR compound. Dashed lines indicate percolation threshold values obtained by RPA measurements. Note that the scaling is twice the scale in Figure 4.

Observing the normalised superharmonic resonance (nSHR) values in Figure 7, the nonlinearity of the resin compound is significantly higher than the nonresin analogue. Utilising the conclusion from the previous studies, the nonlinearity induced by the filler network is enhanced by the resin and is attributed to a more compact network formed in the polymer.

## 4. Conclusions

A systematic study of three variants of SBR matrix filled with either N121, N339, or N550 carbon black grades was performed in order to elaborate the superharmonic resonance effects in terms of filler volume fractions. The filler-induced nonlinearity in the N550 compound is much weaker than the other two counterparts, indicating that the microdispersion of the filler plays a role in the superharmonic resonance effects, as validated by TEM images. Following this, reinforcing resin was introduced in an IR matrix in order to investigate the effects of a compact filler network. The compactness of the filler network leads to the compounds with high resin content to exhibit a significantly high normalised superharmonic resonance, further consolidating the hypothesis that the filler network is indeed inducing a secondary mechanical propagation wave which leads to the nonlinearity effects of filled compounds.

## Figures and Tables

**Table 1:** Recipe for the carbon-black-type series used in this study (all values in parts per hundred rubber).

Ingredients	N121	N339	N550
SSBR ${}^{a}$	100.0	100.0	100.0
Filler	0.0–55.0	0.0–55.0	0.0–75.0
IPPD ${}^{b}$	1.5	1.5	1.5
Zinc Oxide	3.0	3.0	3.0
Stearic Acid	1.0	1.0	1.0
CBS ${}^{c}$	2.0	2.0	2.0
Sulphur	1.4	1.4	1.4

${}^{a}$ Microstructure: styrene content 24%, Vinyl content 34%, molecular weight 472.65 kg/mol, Tg $=-40{\;}^{\circ }$C. ${}^{b}$ *N*-isopropyl-$N^{\prime }$-phenyl-1,4-phenylenediamine. ${}^{c}$ *N*-cyclohexyl-2-benzothiazole sulfenamide.

**Table 2:** Recipe for the reinforcing resin series used in this study (all values in parts per hundred rubber).

Ingredients	Reinforcing Resin
IR ${}^{d}$	100.0
N339	20.0–60.0
Resorcinol	0.0/0.7/4.6
HMMM ^e^	0.0/0.7/4.6
6PPD ${}^{f}$	2.0
TMQ ${}^{g}$	1.0
Zinc Oxide	6.0
Stearic Acid	1.0
Processing Oil	3.0
DCBS ${}^{h}$	1.0
Sulphur	4.0

${}^{d}$ Microstructure: 97% cis, molecular weight 1300 kg/mol, Tg $=-65{\;}^{\circ }$C. ^e^ hexamethoxymethyl-melamine. ${}^{f}$ *N*-(1,3-dimethylbutyl)-*N*${}^{\prime }$-phenyl-p-phenylene diamine. ${}^{g}$ 2,2,4-trimethyl-1,2-dihydroquinoline. ${}^{h}$ *N*,*N*${}^{\prime }$-dicyclohexyl-2-benzothiazole sulfenamide.

## References

[1] Vilgis T.A., Heinrich G., Klueppel M. (2009). Reinforcement of Polymer Nano-Composites: Theory, Experiments and Applications.

[2] Heinrich G., Klüppel M. (2002). Recent Advances in Polymer Science. Filled Elastomers: Drug Delivery Systems.

[3] Payne A.R. (1965). Effect of dispersion on the dynamic properties of filler-loaded rubbers. J. Appl. Polym. Sci..

[4] Schwab L. (2016). Fourier Transform Rheology of Complex, Filled Rubber Materials. Ph.D. Thesis.

[5] Ewoldt R.H., Winter P., Maxey J., McKinley G.H. (2010). Large amplitude oscillatory shear of pseudoplastic and elastoviscoplastic materials. Rheol. Acta.

[6] Ewoldt R.H., McKinley G.H. (2010). On secondary loops in LAOS via self-intersection of Lissajous—Bowditch curves. Rheol. Acta.

[7] Wilhelm M., Reinheimer P., Ortseifer M. (1999). High sensitivity Fourier-transform rheology. Rheol. Acta.

[8] Hyun K., Wilhelm M., Klein C.O., Cho K.S., Nam J.G., Ahn K.H., Lee S.J., Ewoldt R.H., McKinley G.H. (2011). A review of nonlinear oscillatory shear tests: Analysis and application of large amplitude oscillatory shear (LAOS). Prog. Polym. Sci..

[9] Landa P.S., McClintock P.V.E. (2000). Vibrational resonance. J. Phys. A Math. Gen..

[10] Yang J., Sanjuán M.A., Liu H. (2016). Vibrational subharmonic and superharmonic resonances. Commun. Nonlinear Sci. Numer. Simul..

[11] Syed I., Vouagner P., Fleck F., Lacayo-Pineda J. (2019). Nonlinearity in the Mechanical Response of Rubber as Investigated by High-Frequency DMA. Polymers.

[12] Syed I., Klat D., Braer A., Fleck F., Lacayo-Pineda J. (2018). Characterizing the influence of reinforcing resin on the structure and the mechanical response of filled isoprene rubber. Soft Mater..

[13] (2018). ASTM D5992-96(2018) Standard Guide for Dynamic Testing of Vulcanized Rubber and Rubber-Like Materials Using Vibratory Methods.

[14] Schwab L., Hojdis N., Lacayo J., Wilhelm M. (2016). Fourier-Transform Rheology of Unvulcanized, Carbon Black Filled Styrene Butadiene Rubber. Macromol. Mater. Eng..

